# Vertical structures of marine heatwaves

**DOI:** 10.1038/s41467-023-42219-0

**Published:** 2023-10-14

**Authors:** Ying Zhang, Yan Du, Ming Feng, Alistair J. Hobday

**Affiliations:** 1 State Key Laboratory of Tropical Oceanography, South China Sea Institute of Oceanology, Chinese Academy of Sciences, Guangzhou, China; 2 University of Chinese Academy of Sciences, Beijing, China; 3 CSIRO Environment, Crawley, WA Australia; 4 CSIRO Environment, Hobart, TAS Australia

**Keywords:** Physical oceanography, Environmental health, Physical oceanography

## Abstract

A marine heatwave (MHW) is typically defined as an anomalous warm event in the surface ocean, with wide-ranging impacts on marine and socio-economic systems. The surface warming associated with MHWs can penetrate into the deep ocean; however, the vertical structure of MHWs is poorly known in the global ocean. Here, we identify four main types of MHWs with different vertical structures using Argo profiles: shallow, subsurface-reversed, subsurface-intensified, and deep MHWs. These MHW types are characterized by different spatial distributions with hotspots of subsurface-reversed and subsurface-intensified MHWs at low latitudes and shallow and deep MHWs at middle-high latitudes. These vertical structures are influenced by ocean dynamical processes, including oceanic planetary waves, boundary currents, eddies, and mixing. The area and depth of all types of MHWs exhibit significant increasing trends over the past two decades. These results contribute to a better understanding of the physical drivers and ecological impacts of MHWs in a warming climate.

## Abstract (web summary)

The authors identify four main types of vertical structures of marine heatwaves, with different impact depths and spatio-temporal distributions, that are influenced by multiscale ocean dynamical processes.

## Introduction

**Fig. 1:**
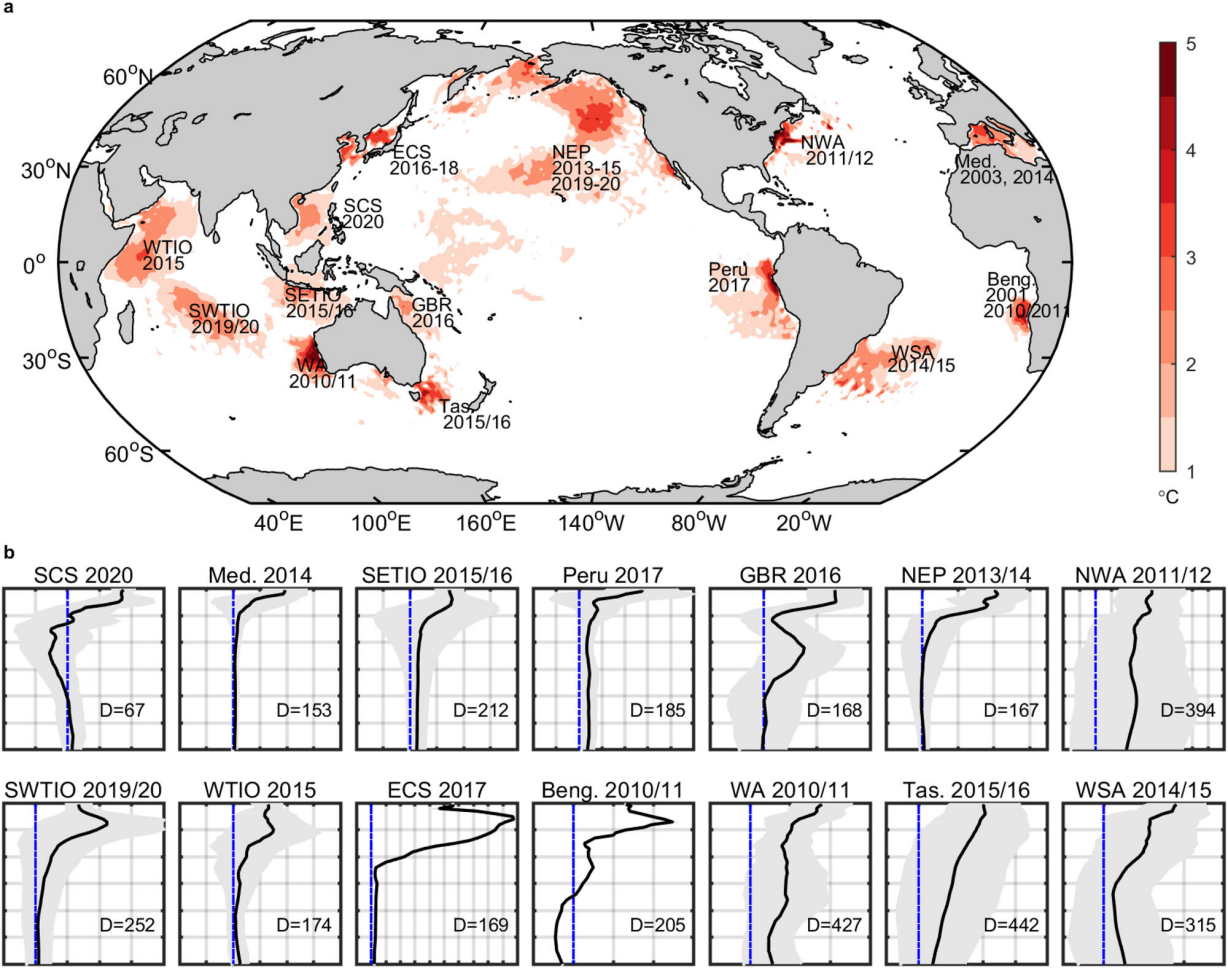
Horizontal and vertical structures of prominent marine heatwaves (MHWs) from the past two decades. **a** Sea-surface temperature anomalies (above 1 °C) on the day of maximum intensity of the prominent MHWs. **b** Average vertical profiles of temperature anomalies for all Argo profiles during the prominent MHWs (black solid lines, °C), with one standard deviation of vertical temperature anomalies for all Argo profiles in a MHW event (gray shading, °C). The average impact depths of all Argo profiles from the MHWs (D, Unit: dbar) are labeled in **b**. Only one Argo profile was found in the 2017 East China Sea and 2010/2011 Benguela MHWs. All events shown in **a** are referenced in Supplementary Table 1. **b** The horizontal axes are the temperature anomalies, with blue dash-dotted lines denoting zeros and grid intervals of 1 °C, and the depths sit on the vertical axes, which start at 0 dbar with 100 dbar intervals. WTIO west tropical Indian Ocean, SWTIO southwest tropical Indian Ocean, SETIO southeast tropical Indian Ocean, WA Western Australia, ECS East China Sea, SCS South China Sea, GBR Great Barrier Reef, NEP northeast Pacific, Tas. Tasman Sea, NWA northwest Atlantic, WSA western South Atlantic, Med. Mediterranean Sea, Beng. Benguela.

Marine heatwaves (MHWs)—periods of anomalously high ocean temperatures—can extend to thousands of kilometers and last for weeks to months^1,2^. Globally, the frequency and duration of MHWs have increased substantially over the past century^3^ and are projected to increase further under continued global warming^4^. Over the past decades, MHWs of record-breaking intensity or/and duration have been observed in the open ocean, marginal seas, and coastal regions (Fig. 1a), with widespread and profound ecological and socio-economic impacts^5–7^. MHWs have catastrophic effects on critical foundation species that play an essential role in the ecological functioning of ecosystems and entire biomes, such as coral bleaching and mortality^8^, and declines in seagrass meadow and kelp forest extent^9,10^. The loss of seagrass, kelp, and coral affects regulating and habitat services derived from marine ecosystems by reducing carbon sequestration and storage, disrupting carbon and nitrogen cycling, and contracting habitats for commercial and iconic species^6,7^. MHWs have also been linked to widespread mortality of invertebrates, pelagic forage species, fish, marine mammals, and fish-eating seabirds, disrupted food webs, shifts in species ranges and abundances, and even loss of biodiversity and genetic diversity^5–7,11^. The mass die-offs and species migrations affect provisioning services provided by marine ecosystems, especially fisheries^6,7^. The provisioning service impact is often accompanied by the cultural service impact associated with tourism and recreational fisheries^6,7^. Thus, ecological impacts of MHWs range from loss of seagrass, kelp, and coral and widespread mortalities to ecosystem reconfigurations, affecting habitat, regulating, provisioning, and cultural ecosystem services globally. Furthermore, MHWs can propagate impacts through remote connections, contributing to severe drought, heavy precipitation, or terrestrial heatwave events^5^. These impacts warn us that the effects of MHWs are not limited to the ocean surface, but extend into the atmosphere and the deep ocean.

The formation, persistence, and decay of MHWs are driven by atmospheric and/or oceanic processes, including changes in air-sea heat flux, horizontal/vertical heat advection, and lateral/vertical mixing^12–14^. Changes in air-sea heat flux result from the gain or loss of shortwave and longwave radiation as well as latent and sensible turbulent heat fluxes, associated with changes in cloud cover, wind speed, surface level relative humidity, and surface air temperature^15,16^. Changes in horizontal heat advection can be driven by anomalous geostrophic and Ekman currents, anomalous temperature gradients, or mesoscale eddies^17,18^. Changes in vertical heat advection are due to thermal stratification changes or upwelling/ downwelling processes^19,20^. Lateral and vertical mixing is related to horizontal diffusive flux and vertical turbulent flux^21^. These processes vary widely across MHWs and depend on where and when MHWs occur. MHWs are also associated with large-scale climate modes, such as El Niño-Southern Oscillation (ENSO), Indian Ocean Dipole (IOD), North Atlantic Oscillation (NAO), Pacific Decadal Oscillation (PDO), Southern Annular Mode (SAM), which modulate ocean temperatures locally or remotely through atmospheric and oceanic teleconnections (e.g., atmospheric or oceanic Kelvin and Rossby waves)^22^.

MHWs with an expression at the ocean surface have been widely studied, partly due to the availability of satellite-observed sea surface temperature (SST) data since 1981. Recently, several studies illustrated that MHWs are not confined to the surface layer but extend to deeper waters in the East Australian Current System^23,24^. They divided MHWs into shallow, intermediate, and deep events according to their vertical extensions. The shallow MHWs were confined to the surface layer, while the mean temperature profiles of intermediate and deep MHWs show greater warming anomalies below the mixed layer^23^. The deepest MHWs are driven by advection, while shallower MHWs are dominated by surface flux^24^. However, it is notable that some shallow and intermediate events have cooling anomalies beneath the warming anomalies, especially the shallow events. Moreover, not all intermediate and deep events have the maximum warming anomalies in the subsurface. These studies imply that surface MHWs have different depths of vertical extension as well as shapes of vertical structures. Still, little is known about the vertical structure of MHWs in the global ocean, and the typical characteristics of the vertical structure of global MHWs remain to be explored. The T/S vertical profiles of Argo floats with a depth ranging from 0–2000 dbar provide an opportunity to explore the vertical structure of MHWs in the global ocean. Therefore, our goals were to identify the vertical structures of global MHWs and their vertical penetration depths, and to explore the spatiotemporal characteristics of MHWs with different vertical structures.

## Results

### Characteristics of MHWs with different vertical structures

**Fig. 2:**
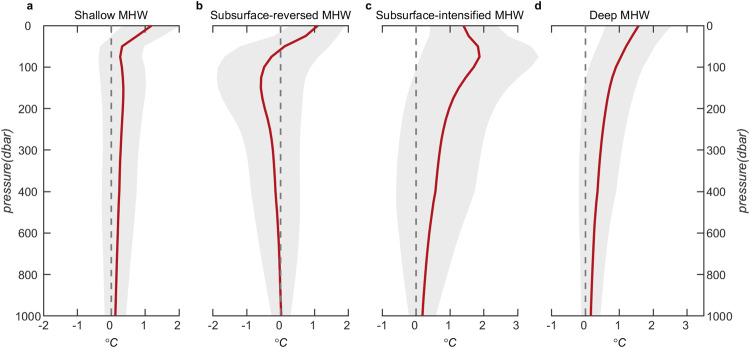
Composites of vertical temperature anomalies for different types of marine heatwaves (MHWs). **a** Shallow MHWs; **b** subsurface-reversed MHWs; **c** subsurface-intensified MHWs; and **d** deep MHWs. The shading represents one standard deviation of the vertical temperature anomalies for each type of MHW (Unit: °C).

**Fig. 3:**
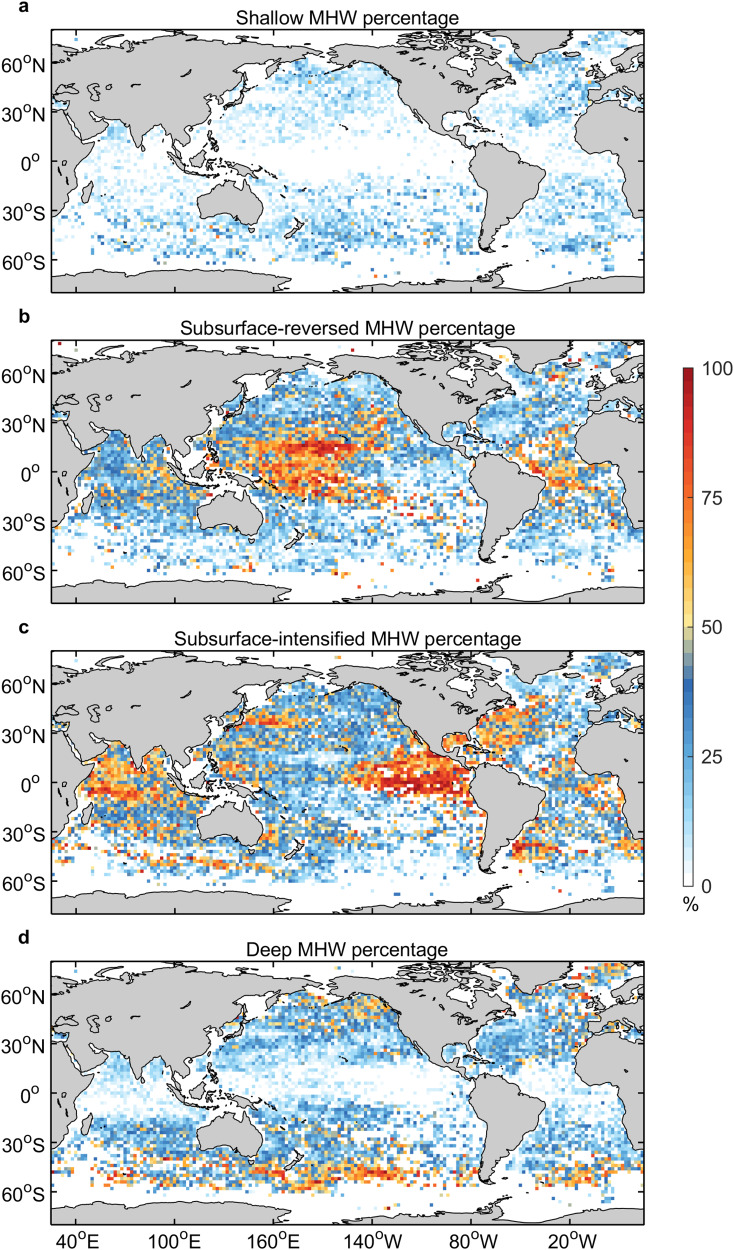
Percentage of marine heatwave (MHW) days with different vertical structures in the total days of all types of MHWs. **a** Shallow MHWs; **b** subsurface-reversed MHWs; **c** subsurface-intensified MHWs; and **d** deep MHWs. The shading is the mean of all percentages, which is greater than one standard deviation, derived from the bootstrap method.

**Fig. 4:**
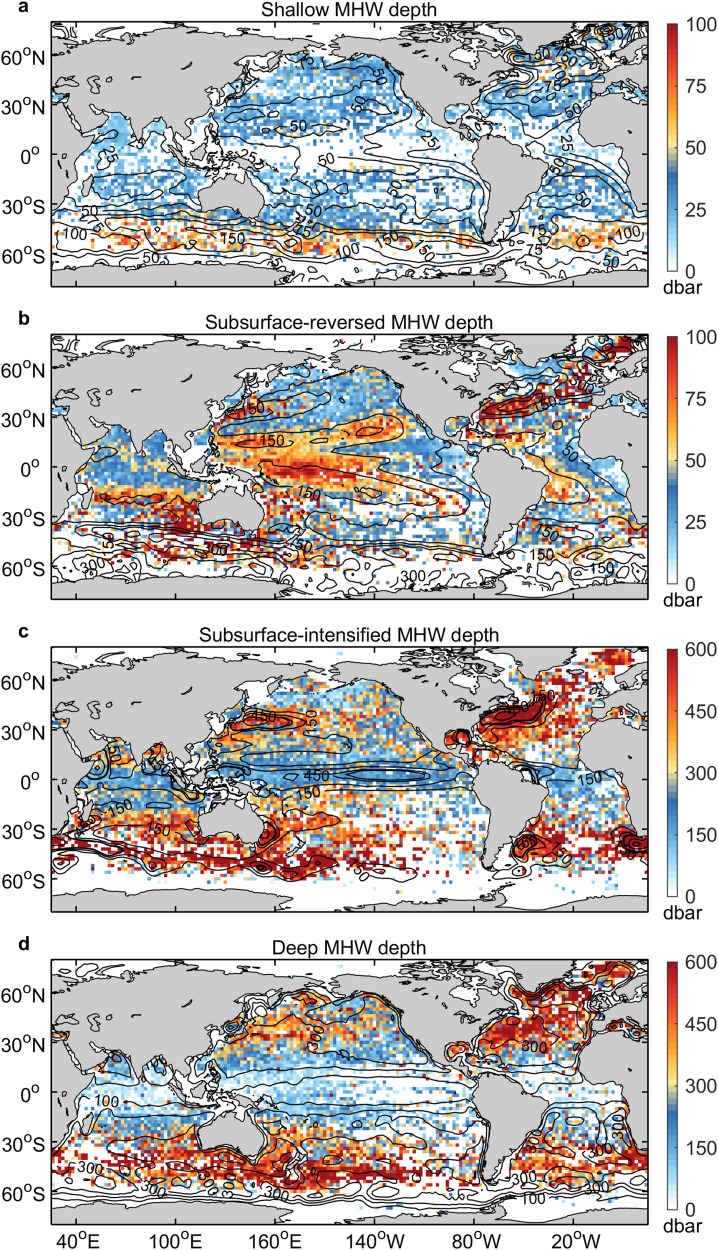
Spatial distribution of the mean impact depths for different types of marine heatwaves (MHWs). **a** Shallow MHWs; **b** subsurface-reversed MHWs; **c** subsurface-intensified MHWs; and **d** deep MHWs. The annual mean mixed-layer depth (contours, dbar), thermocline depth (contours, dbar), eddy kinetic energy (contours, m^2^ s^−2^), and total days of eddy occurrence per year (contours, days) are superimposed in **a**–**d**, respectively.

We grouped MHWs into four types according to their vertical structures (see Methods for more details): (1) shallow MHWs, where warming is confined to the surface layer and decreases rapidly with depth (Fig. 2a); (2) subsurface-reversed MHWs, which have warming at the surface and anomalous cooling beneath this surface warming (Fig. 2b), e.g., mean temperature anomaly profile of the 2020 South China Sea MHW (Fig. 1b); (3) subsurface-intensified MHWs, which exhibit maximum warming anomalies in the subsurface layers (Fig. 2c), e.g., mean temperature anomaly profile of the 2019/20 southwest tropical Indian Ocean MHW (Fig. 1b); (4) deep MHWs, which display surface warming anomalies that decay slowly with depth (Fig. 2d), e.g., mean temperature anomaly profiles of the 2010/11 Western Australia, the 2011/12 northwest Atlantic, the 2014/15 western South Atlantic, the 2013–15 northeast Pacific, the 2015/16 Tasman Sea, and the 2016 Great Barrier Reef MHWs (Fig. 1b).

It is evident that these four types of MHWs have different spatial distributions (Fig. 3). Shallow MHWs occur mostly in the middle-high latitudes, with an overall small proportion compared to other types. Subsurface-reversed and subsurface-intensified MHWs are distributed across the global ocean. Notably, large spatial variations are prevalent in the proportion of these two types of MHWs. Hotspots (>50% of one type) occur mainly in tropical oceans, where oceanic planetary wave processes are active and associated with climate modes. Oceanic planetary wave processes drive thermocline fluctuations, which in turn affect the subsurface signals of MHWs (Supplementary Fig. 2). In the tropical Pacific, the hotspots of subsurface-reversed MHWs are in the central basin, while the subsurface-intensified MHWs are in the east and west of the basin. This is consistent with the relationship between surface and subsurface temperature anomalies, with negative correlations in the central tropical Pacific and positive correlations in the east and west (Supplementary Fig. 3). This implies that the physical processes that dominate the formation, evolution, and decay of MHWs are regionally diverse. In the tropical Indian Ocean, the proportion of subsurface-intensified MHWs is higher than subsurface-reversed MHWs, especially in the western basin. In the tropical Atlantic Ocean, the proportion of subsurface-intensified is comparable to that of subsurface-reversed MHWs, but the spatial distribution is the opposite, with a higher proportion of subsurface-intensified in the east and a higher proportion of subsurface-reversed in the west. Deep MHWs appear primarily in the subtropical-subpolar regions (30°N/S–60°N/S), accounting for a large proportion of all events.

The impact depth of MHWs (see Methods for definition) is an important indicator in evaluating their vertical characteristics and exploring their impact on marine organisms and ecosystems. The impact depths of these four types of MHWs differ in their global distribution. Both shallow MHWs and subsurface-reversed MHWs tend to have shallow impact depths, while both subsurface-intensified MHWs and deep MHWs tend to have deep impact depths (Fig. 4). As the warming of shallow MHWs is limited to the surface, their depths are shallow in the majority of oceanic regions, except at high latitudes where they are relatively deep, with a global average depth of 32 ± 16 dbar (Fig. 4a). A correlation coefficient of 0.56 between the spatial distributions of the shallow MHW depths and mixed-layer depths (MLDs) indicates a connection between them. The subsurface-reversed MHWs tend to have shallow impact depths, with a global mean depth of 57 ± 38 dbar (Fig. 4b). To a certain extent, the spatial distribution of subsurface-reversed MHW depths follows that of the thermocline depths, with a correlation coefficient of 0.39. The deeper subsurface-reversed MHWs are found in the western basins of the Pacific and Atlantic Oceans, while the shallower MHWs are found in the eastern basins, due to the west-to-east upward-tilted thermocline in the Pacific and Atlantic Oceans. In the Indian Ocean, the shallower subsurface-reversed MHWs are located in the thermocline dome region of the southwest tropical Indian Ocean. The subsurface-intensified MHWs tend to have deep impact depths, with a global mean depth of 348 ± 190 dbar (Fig. 4c). The deeper subsurface-intensified MHWs are found in the subtropical gyres associated with deeper mixed layer and thermocline of the subtropical convergence zones, whereas the shallower MHWs are found in the tropical oceans related to the shallower mixed-layer and thermocline of the tropical divergence zones. In particular, the eastern tropical Pacific and western tropical Indian Ocean, which are hotspots for subsurface-intensified MHWs, have shallow mixed-layer and thermocline depths. The spatial distribution of subsurface-intensified MHW depths correlates with that of MLDs at 0.43 and thermocline depths at 0.35. For the deep MHWs, their depth distribution resembles that of subsurface-intensified MHWs, being relatively deep in the subtropical to subpolar regions and shallow in the tropical oceans, with a global average depth of 316 ± 202 dbar (Fig. 4c, d). The spatial distribution of deep MHW depths shows some similarities to that of MLDs with a correlation coefficient of 0.54 (Fig. 4a, d). In the North Pacific, North Atlantic, southeast Indian, and Southern Oceans, the subsurface-reversed, subsurface-intensified, and deep MHWs all have greater impact depths (Fig. 4b–d), which is associated with strong currents as well as active mesoscale eddies. These results suggest an important role of oceanic stratification in the distribution of MHW depths, involving thermal and dynamical processes of oceanic heat absorption and transfer.

### Potential drivers for different vertical structures of MHWs

These four types of MHW have different spatial distributions and vertical depths, which may depend on the regionally dominant physical processes not only in the mixed layer but also in the thermocline. The potential drivers for MHWs are now explored through the analysis of a mixed-layer heat budget (see Methods for details).

### Tropical oceans

**Fig. 5:**
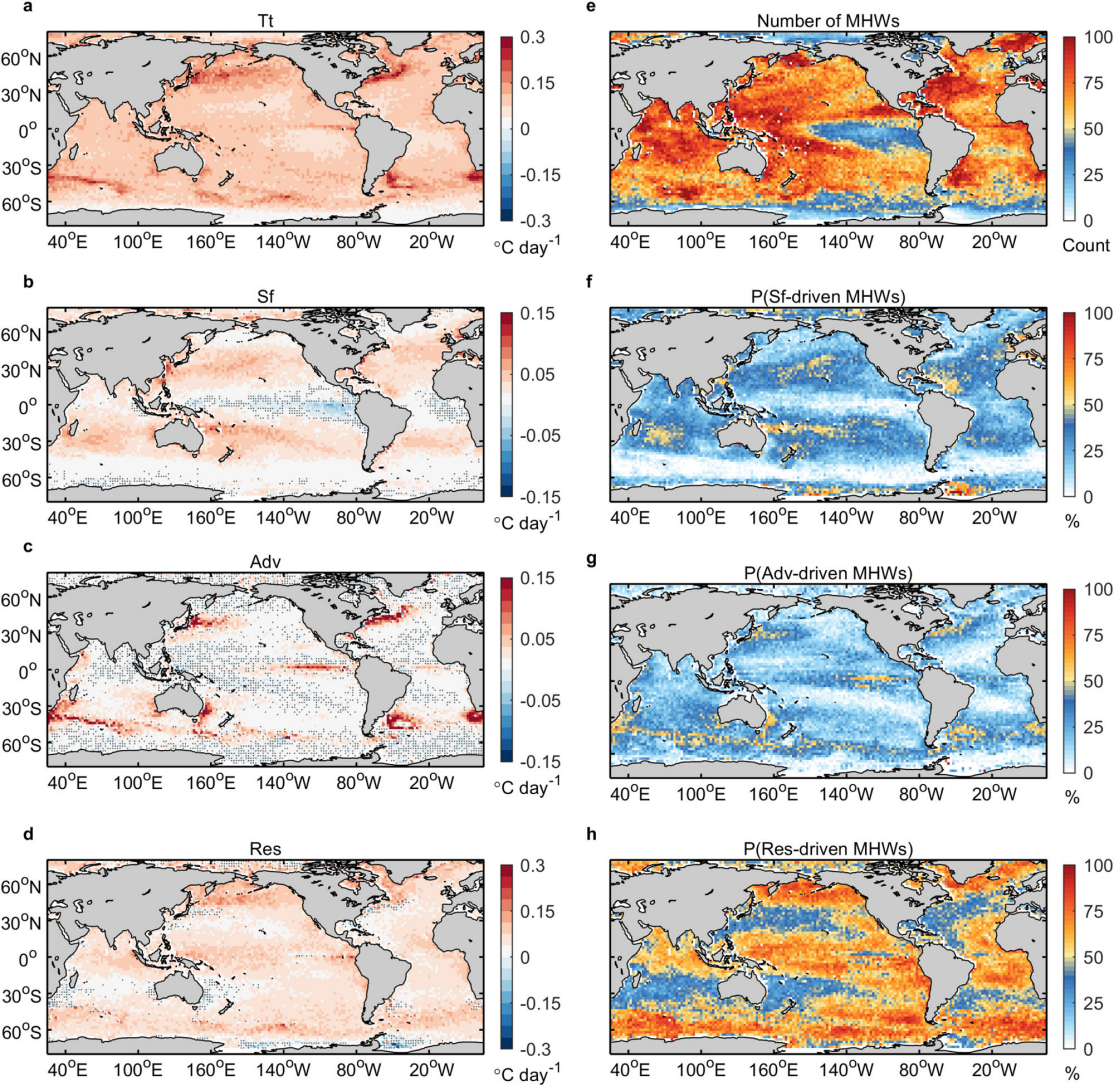
Heat budgets for marine heatwaves (MHWs). **a**–**d** Composite of temperature tendency, surface flux forcing, horizontal heat advection, and residual terms during the development phase of MHWs, respectively. **e** Total number of MHW events. **f**–**h** Percentage of surface flux-driven MHWs, advection-driven HMWs, and residual-driven MHWs to the total number of MHWs, respectively. The dotted areas on (**a**–**d**) show that the composite mean is smaller than one standard error.

In the tropical oceans, ocean dynamical processes, especially vertical processes, play a crucial role in the formation and evolution of MHWs (Fig. 5). When the trade winds weaken in the tropical Pacific, the anomalous winds drive the downwelling Kelvin waves that propagate eastward and deepen the thermocline in the eastern Pacific, whereas the upwelling Rossby waves propagate westward and lift the thermocline in the western Pacific^25–27^. These processes facilitate the formation of subsurface MHWs, with strong subsurface warming in the eastern tropical Pacific forming subsurface-intensified MHWs, while strong subsurface cooling in the central tropical Pacific resulting in subsurface-reversed MHWs. The opposite situation is seen when the trade winds intensify over the tropical Pacific, contributing to the subsurface-intensified MHWs in the western tropical Pacific^28^. In the tropical Atlantic and Indian Oceans, similar phenomena occur in response to surface wind anomalies^29,30^. Several prominent MHWs with remarkable subsurface warming or cooling have been observed in the tropics, e.g., the 2010/11 Benguela^31^, the 2017 Peru^19^, the 2015 west tropical Indian Ocean, the 2015/16 southeast tropical Indian Ocean^32^, and the 2019/20 southwest tropical Indian Ocean MHWs^20^, closely linked to the thermocline modulation caused by oceanic downwelling or upwelling planetary waves. Moreover, higher correlations of 0.59 and 0.78 are found in the tropical oceans between the depths of maximum cooling and warming of MHWs and the depths of the thermocline, compared to global correlations of 0.46 and 0.55 (Supplementary Fig. 2), respectively. Thus, oceanic planetary waves can play an important role in shaping the vertical structure of MHWs in tropical oceans.

### Subtropical oceans

In the subtropical oceans, air-sea heat flux anomalies associated with high-pressure systems, consisting primarily of anomalous solar radiation and latent heat flux due to reduced cloud cover and wind speed^15,16,33,34^, are the main factors of the formation and evolution of MHWs (Fig. 5). Elzahaby et al.^24^ suggested that the surface flux-driven MHWs have shallower depth and shorter duration, compared to the advection-driven MHWs. This is because the air-sea heat flux is mainly absorbed by the upper ocean and there is rapid feedback with the atmosphere. Therefore, shallow MHWs, characterized by strong warming confined to the surface layer, occur mostly in the subtropical oceans, but represent only a small proportion of events (Fig. 3). Furthermore, a shallow MHW could be the start or end of a deep or subsurface MHW event when atmospheric forcing or oceanic processes are initiating or relaxing.

Surface warming can penetrate into the subsurface through subduction (Ekman pumping or lateral advection), favoring the generation of deep MHWs (Fig. 3), such as the 2013–15 northeast Pacific MHWs, known as “the Blob”^35,36^, and the 2019–20 northeast Pacific MHWs, “Blob 2.0”^37^. Even in surface flux-driven MHWs, the warming signature can extend to the subsurface if the ocean dynamical processes are also in favor, such as the 2011/12 northwest Atlantic^34,38^ and 2014/15 western South Atlantic MHWs^16^.This explains the prevalence of deep MHWs in the subtropical oceans.

### High-latitude oceans

High-latitude oceans are important regions for the formation of water masses as part of ocean ventilation, particularly in the North Atlantic and Southern Oceans^39^. In the Southern Ocean, heat advection by the Antarctic Circumpolar Current (ACC) and mesoscale eddies are responsible for the development of MHWs (Fig. 5). Thus, deep MHWs are dominant in the ACC region (Fig. 3). The ocean vertical processes, possibly associated with upwelling and mixing anomalies^40^, dominate the generation of MHWs south of the ACC. A similar situation occurs in the high latitudes of the North Pacific and Atlantic. Modulated by the ocean vertical processes, the warming of MHWs can extend into the deep ocean, forming deep MHWs. Furthermore, the subducted water masses carrying anomalous cooling or warming signals as well as mode-water eddies^41,42^ contribute to the formation of subsurface-reversed and subsurface-intensified MHWs in the high-latitude oceans.

### Western boundary current regions

Fast, narrow, and deep western boundary currents are an important component of the subtropical gyres and transport large amounts of heat poleward^43^. Enhanced horizontal heat advection is an essential driver for the formation and evolution of MHWs in these regions (Fig. 5). As a result, deep MHWs occur frequently here (Fig. 3), such as the 2015/16 Tasman Sea MHW where the warming into the deep ocean was caused by enhanced heat advection^18^.

Western boundary currents are eddy-rich zones with high eddy kinetic energy and temperature fronts^43^. The eddies play an important role in shaping the vertical structure of MHWs, especially in the western boundary currents regions (Supplementary Figs. 4–5). A larger proportion of anticyclonic eddies occur with shallow and deep MHWs, with stronger warming, compared to cyclonic eddies (Supplementary Fig. 5). Nevertheless, a larger proportion of cyclonic (anticyclonic) eddies than anticyclonic (cyclonic) eddies is detected in subsurface-reversed (subsurface-intensified) MHWs, with stronger subsurface cooling (warming). Furthermore, there are two main types of eddies observed in the global ocean: surface eddies, where the eddy core is near the surface, and subsurface eddies, where the eddy core is near the thermocline^41,42,44^. They have different impacts on the vertical structure of the MHWs, with surface anticyclonic (cyclonic) eddies typically enhancing (weakening) surface warming of MHWs and subsurface anticyclonic (cyclonic) eddies usually reinforcing subsurface warming (cooling) (Supplementary Fig. 5).

### Eastern boundary current regions

In most subtropical gyres, eastern boundary currents carry cold water towards the equator with the upwelling of cool water along the coast^45^. In these regions, the formation of MHWs is largely driven by oceanic forcing, especially vertical processes, while atmospheric forcing also contributes to the generation of MHWs (Fig. 5). The coastal downwelling Kelvin waves, some originated in the equatorial regions, deepen the thermocline, suppress the upwelling of cool water, and enhance the poleward advection of warm water^22,46,47^. The establishment of positive air-sea feedback can weaken the alongshore winds and contribute to coastal warming^22,46,47^. For example, the coastal Kelvin waves and local wind anomalies are crucial in driving the 2010/11 Benguela^31^ and the 2017 Peru MHWs^19^. Therefore, the four types of MHWs appear in these regions in equal proportions due to these complex air-sea coupling processes.

In contrast, the Leeuwin Current in the eastern boundary of the southern Indian Ocean brings warm water poleward and has high eddy kinetic energy^48–50^. The enhanced poleward heat advection of the Leeuwin Current is an important contributor to the development of MHWs in the southeast Indian Ocean (Fig. 5), favoring the deep MHWs that prevail here (Fig. 3). The formation of MHWs in this region is also supported by atmospheric forcing. For instance, the 2010/11 Western Australia MHW with deep warming was caused by a combination of strengthened poleward advection of a record-strength Leeuwin Current and positive anomalous air-sea heat flux into the ocean^17^. Moreover, the convergence and divergence of upper ocean warm water driven by oceanic planetary waves and mesoscale eddies and heat advection by undercurrent^48–50^ can affect the vertical structure of MHWs in these regions.

### Long-term trends of four types of MHWs

**Fig. 6:**
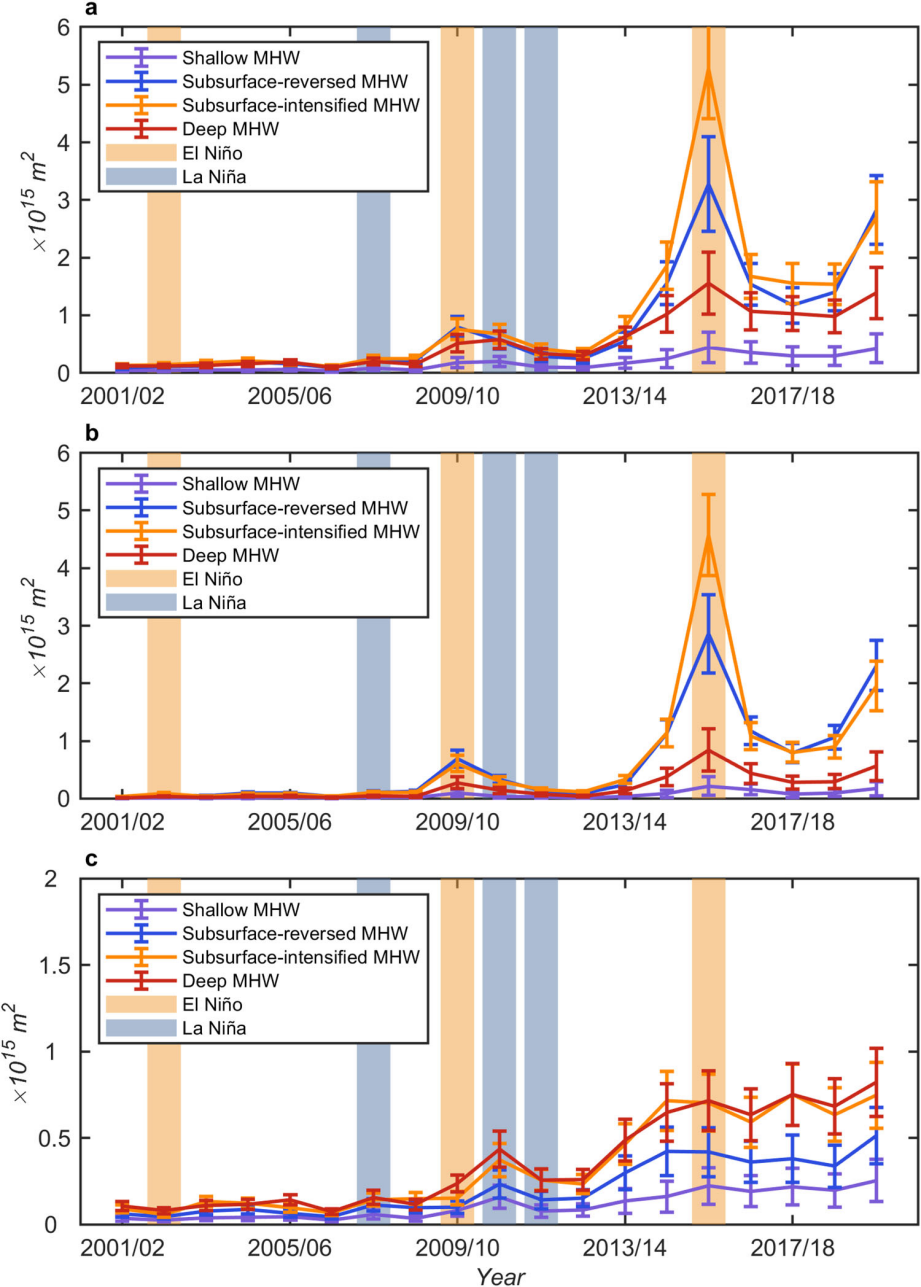
Globally and regionally integrated time series of the total area of annual marine heatwave occurrence. **a** Global; **b** low-latitude region (30°S-30°N); and **c** medium-high-latitude region (80°S/N–30°S/N). The vertical error bars are standard deviations of all values derived using the bootstrap method that represents the uncertainty due to the uneven distribution of Argo profiles. Given the lead-lag effect of El Niño-Southern Oscillation, the annual average is taken from July of the first year to June of the second year.

The ocean areas experiencing MHWs increased significantly during the Argo era (2001–2020), with the greatest increase in the occurrence area of the subsurface-intensified MHWs (1.52 × 10^14^ m^2^ yr^−1^, *p* < 0.01) and the least increase in the occurrence area of the shallow MHWs (2.13 × 10^13^ m^2^ yr^−1^, *p* < 0.01). The occurrence area of the subsurface-reversed MHWs showed a rising trend of 1.27 × 10^14^ m^2^ yr^−1^ over 2001–2020, followed by the deep MHW, with an increasing trend of 7.38 × 10^13^ m^2^ yr^−1^ (*p* < 0.01; Fig. 6a). The long-term warming of the global ocean dominates the increasing trend of MHWs, whereas the changes in internal temperature variability play a secondary role^3^. For example, the positive phase of the PDO favored the occurrence of northeast Pacific MHWs over recent years^15,51,52^, which might contribute to the increasing trend of MHWs during the Argo era. However, the Argo record is too short to distinguish decadal to multidecadal climate variability, such as PDO and Atlantic Multidecadal Oscillation (AMO), from long-term trends.

The time series of ocean areas experiencing MHWs also exhibited clear interannual variability related to climate mode variabilities^22^. This variability seems related to ENSO events, the dominant global climate mode. The occurrence of MHWs peaked in the global ocean during El Niño events (2009/10 and 2015/16). However, there was no peak detected in 2002/03 El Niño, probably because 2002/03 El Niño was not as strong as 2009/10 and 2015/16 El Niño events. The peak in 2019/20 might be associated with the extreme positive IOD in 2019 on record and the resulting Indian Ocean basin-wide warming the following year^53,54^. In addition, the northeast Pacific MHWs in 2013–15 and 2019–20 were associated with North Pacific Gyre Oscillation (NPGO) and PDO, contributing to the peaks in global MHWs^15,51,52^.

In tropical oceans, the interannual variabilities of MHWs are more remarkable than those in the middle-high latitudes (Fig. 6b, c), related to climate modes such as ENSO and IOD. This indicates that MHWs respond to the redistribution of ocean heat content associated with internal climate variabilities, particularly subsurface-reserved and subsurface-intensified MHWs. Notably, compared with MHWs in the tropical oceans, the MHWs in the middle-high latitudes have significant increasing trends, while relatively weak interannual variabilities, especially subsurface-intensified and deep MHWs (Fig. 6c). In the middle-high latitudes, both the surface and subsurface temperature, and thus the ocean heat content, have shown significant warming over the past decades. Subsurface warming appears to result mostly from heat entering via subduction and spreading laterally from the ventilation zones of subtropical mode waters^55^ and Antarctic mode waters^56^. Correspondingly, MHWs store large amounts of ocean heat content in the middle-high latitudes. In addition, the subsurface warming in the middle-high latitudes can be transported into the tropics by the subtropical gyres and meridional overturning circulations^57^. In general, the depths of all types of MHWs show increasing trends over the Argo era (Supplementary Fig. 6). The increases in the depths of MHWs at mid-high latitudes are largely attributed to an increase of upper ocean heat content, particularly in subsurface-intensified and deep MHWs. In the western tropical Pacific and tropical Atlantic Oceans, the changes in the variance of upper ocean heat content contribute to the increased depths of subsurface MHWs (Supplementary Fig. 7).

## Discussion

This study detected the spatiotemporal distribution of MHWs characterized by four types of vertical structures using Argo profiling floats from 2001–2020. Although subsurface measurements are available at relatively low temporal resolution, they provide a valuable opportunity to understand the diversity of vertical structures of MHWs. These types of MHWs reveal the important role of oceanic multiscale dynamical processes in shaping the vertical structure of MHWs, involving oceanic planetary waves, large-scale currents, eddies, and mixing.

Both the area of occurrence and depth of four types of MHWs show increasing trends over the Argo era in response to rising global ocean temperatures^3^. The ocean has absorbed and stored about 90% of the global warming heat entering Earth’s climate system^58,59^. Thus, a change in ocean temperature is a crucial indicator of climate change. However, the changes in ocean temperature are spatially heterogeneous and depend on ocean dynamical processes (e.g., subtropical gyres, meridional overturning circulation, mixing), climate modes (e.g., ENSO, PDO, AMO), as well as response times (e.g., the ocean mixed-layer adjusts quickly, the deep ocean responds slowly)^57,59–61^. This influences the distribution and variability of different types of MHWs. The dominant processes of ocean temperature change differ across the regions. In the subtropical oceans, the downward displacement of isopycnals (heaving) due to wind-driven warm water convergence dominates the subsurface warming^56^; while in the tropical oceans, the upward displacement of isopycnals caused by wind-driven warm water divergence (heaving) and cooling trends on isopycnals propagating along subtropical gyres from mid-high latitudes resulting from migration of the outcropping lines (spiciness) combine to cause the subsurface cooling^62^. For different response times, the deep ocean (>700 m) temperature has continued to warm, while the upper ocean (<300 m) temperature appears to have stabilized during the global warming hiatus^60,61^. In particular, south of the ACC in the Southern Ocean, the surface temperature showed a cooling trend while subsurface temperature showed a warming trend^63^. This indicates that fast mixed-layer response and slow deep ocean response influence the pattern of ocean warming, particularly the vertical distribution of heat^60,61^. Thus, the vertical structures of MHWs represent an important aspect of future studies of ocean warming.

MHWs have now emerged as one of the major challenges to marine ecosystems and the sustainability of marine resources due to their negative impacts on many marine organisms and ecosystems^5–7^. As the MHWs extend warming to the depths, the impacts on marine organisms and ecosystems are not limited to the surface ocean^64^. Notably, subsurface MHWs have stronger temperature anomalies than on the surface, which means that the impact of MHWs on the marine ecosystem should not be evaluated from the SST alone. Therefore, the impacts of MHWs on marine organisms and ecosystems, especially mesopelagic species, deserve further investigation. To improve predictions of MHWs in a future warmer ocean and provide managers of fisheries, aquaculture, and conservation with forecasts to support mitigation strategies, understanding the physical processes and climate drivers of MHWs will be critical^65,66^.

High-resolution numerical models are important tools for understanding these processes. However, they still face challenges in accurately simulating boundary currents, eddies, submesoscale processes, coastal processes, and air-sea exchanges that affect SST patterns or variabilities^67,68^. Thus, improving the spatial coverage and temporal resolution, and maintaining a long-term record of observations could contribute to a greater understanding of the physical properties and drivers of MHWs from the surface to the deep ocean. Long-term reliable observational data sets can also provide improvements for the development of regional or global ocean and climate models, which can likewise help to improve the assessment of the physical properties and drivers of MHWs.

## Methods

### Definition of marine heatwave

Following Hobday et al.^2^, a MHW is defined as an anomalous warm event at the sea surface, lasting five or more days, when SSTs exceed the 90th percentile of a 30-year average seasonal climatology (1982–2011). A fixed-baseline definition of MHWs is used in this study, consistent with most other relevant studies, as it better reflects the destructive impacts of MHWs on marine ecosystems, which typically have slow adaptations to warming temperatures, and multiscale interactions between the ocean and the climate systems^69^. This definition is applied to the National Oceanic and Atmospheric Administration (NOAA) Optimum Interpolation (OI) SST V2.1 global daily gridded SST data^70^ over 1982–2021 to detect global MHWs. Here, we upscaled the high-resolution SST data from 1/4° grid to 2° grid, considering the sparsity of subsurface measurements.

To obtain MHWs’ vertical structure, the T/S vertical profiles of Argo floats from 2001 to 2020 are used, which are obtained from the China Argo Real-time Data Center (CARDC)^71^. Argo floats typically sample and measure pressure, salinity, and temperature from the sea surface (~5 dbar) to 2000 dbar on 10-day cycles. Argo floats record temperature and salinity at ~70 depth levels with a vertical sample spacing of 10–25 dbar above 500 dbar and gradually increasing to 50 dbar below 500 dbar. To date, the Argo program has almost 4000 active profiling floats distributed over most of the global ocean and has collected >2 million, high-quality profiles. All available quality-controlled T/S data are mapped into a 2° grid resolution, consistent with SST data, and linearly interpolated onto 58 depth levels. The spatial distribution of the number of Argo vertical profiles within 2° × 2° bins from 2001–2020 is shown in Supplementary Fig. 1. Individual profiles of temperature anomalies, Ta(z) are calculated by subtracting the seasonal climatological profiles from the CSIRO Atlas of Regional Seas (CARS) 2009. The vertical structure of MHWs is defined as the average temperature anomalies of the Argo float profiles falling within the MHW time intervals. There are more than 20 Argo float profiles that recorded these MHW events in most of the ocean, which provides sufficient sampling for the analysis below (Supplementary Fig. 1).

### Definition of MLD

MLD is defined as the depth where potential density differs from the 10-dbar value by 0.03 kg m^−3^ ^72^.

### Definition of thermocline depth

Thermocline depth is the depth of the maximum vertical temperature gradient^73^.

### Mixed-layer heat budget

A mixed-layer heat budget can be used to elucidate the physical drivers of MHWs^13^. The formula is as follows:

1 $$\frac{\partial T}{\partial t}=\frac{Q-{Q}_{d}}{h\rho {C}_{p}}-\mathop{{{{{{\bf{u}}}}}}}\limits^{ \rightharpoonup }\cdot \, \nabla T+{{\mbox{Res}}}$$

Where $T$ is the temperature in the surface mixed layer, $t$ is time, $\mathop{{{{{{\bf{u}}}}}}}\limits^{ \rightharpoonup }=(u,v)$ is the two-dimensional horizontal velocity vector, $\nabla$ is the horizontal gradient operator, $Q$ is the sum of air-sea heat fluxes, including shortwave radiation, longwave radiation, sensible heat flux, and latent heat flux, ${Q}_{d}$ is the shortwave radiation penetrating below the mixed layer, $\rho$ is the seawater density, ${C}_{p}$ is the specific heat capacity of seawater, $h$ is the MLD. The term from the left-hand side is temperature tendency, the first term from the right-hand side is surface flux forcing, the second is horizontal advection, and the third is residual, including mainly vertical entrainment and mixing. In this study, the contributions of the physical processes to the development of MHWs are assessed for the period 1993–2020. The development phase of MHWs refers to the day from the start of MHWs to the day when MHWs reach their maximum intensity. The temperature tendency is computed from NOAA OISST; the surface flux forcing is computed from NCEP-DOE Reanalysis 2 daily air-sea heat fluxes and GLORYS12V1 MLD; the horizontal advection is calculated by combining OSCAR horizontal currents and NOAA OISST at a horizontal resolution of 0.25° and then interpolating to a 2° × 2° grid.

### Definition of vertically cumulative temperature anomaly

The vertically cumulative temperature anomaly represents a scaled version of the heat content anomaly in the MHWs^23^, which is formulated as follows

2 $${{\mbox{CTa}}}({p}_{n})=\mathop{\sum }\limits_{0}^{{p}_{n}}{{\mbox{Ta}}}(p)\Delta p$$

where *n* = 1, 2, 3, … *p*(Ta = 0). The maximum vertically cumulative temperature anomaly is ${{\mbox{MCTa}}}=\mathop{\sum }\nolimits_{0}^{p({{\mbox{Ta}}}=0)}{{\mbox{Ta}}}(p)\Delta p$.

### Definition of four types of MHW vertical structures

The impact depth of MHW (IDMHW) is defined here as the depth at which 85% of the maximum vertically cumulative temperature anomaly (0.85 × MCTa) is located^23^.

3 $${{\mbox{IDMHW}}}=p({{\mbox{CTa}}}({p}_{n})=0.85\times {{\mbox{MCTa}}})$$

A MHW is classified as a shallow MHW when its impact depth is shallower than the minimum depth of [MLD, 100 dbar]. A MHW is classified as a deep MHW when its impact depth is deeper than the minimum depth of [MLD, 100 dbar]. In addition, shallow and deep MHWs display surface warming anomalies that decay with depth without strong subsurface warming anomalies and cooling anomalies (i.e., the subsurface warming/cooling anomalies should be below/above 50th percentiles of subsurface warming/cooling anomalies). A MHW is classified as a subsurface-reversed MHW if it has an anomalous cooling beneath the surface warming and the subsurface cooling anomaly (${{{\mbox{Ta}}}}_{\min }$) is below its 50th percentile. A MHW is classified as a subsurface-intensified MHW if it has the maximum warming anomaly in the subsurface layer and the difference between maximum subsurface warming anomaly and sea surface warming anomaly (${{{\mbox{Ta}}}}_{\max }-{{\mbox{SSTa}}}$) exceeds its 50th percentile.

### Estimation of eddy-induced temperature anomaly

Mesoscale Eddy-Trajectory Atlas products including location, edge contour, amplitude, radius, rotation speed, and polarity of each identified eddy, are derived from AVISO. The temperature anomaly induced by eddy is the difference between the temperature anomaly with eddy and the average temperature anomaly without eddy, which is estimated as follows,

4 $${{{\mbox{Ta}}}}_{{{\mbox{AE}}}}^{{\prime} }={{{\mbox{Ta}}}}_{{{\mbox{AE}}}}-\bar{{{\mbox{Ta}}}}$$

5 $${{{\mbox{Ta}}}}_{{{\mbox{CE}}}}^{{\prime} }={{{\mbox{Ta}}}}_{{{\mbox{CE}}}}-\bar{{{\mbox{Ta}}}}$$

${{{\mbox{Ta}}}}_{{{\mbox{AE}}}}$ is the temperature anomaly overlaid on anticyclonic eddy, ${{{\mbox{Ta}}}}_{{{\mbox{CE}}}}$ is the temperature anomaly overlaid on cyclonic eddy, and $\bar{{{\mbox{Ta}}}}$ is the average temperature anomaly without the occurrence of eddy. Surface anticyclonic/cyclonic eddy refers to the eddy with the strongest warming/cooling anomaly in the surface layer, and subsurface anticyclonic/cyclonic eddy refers to the eddy with the strongest warming/cooling anomaly in the subsurface ocean.

### Supplementary information

Supplementary Information

## Data Availability

We have used publicly available data only. NOAA OISST V2.1 sea surface temperature data was provided by NOAA/OAR/ESRL PSD (Boulder, CO, USA) from their website (https://www.ncei.noaa.gov/data/sea-surface-temperature-optimum-interpolation/v2.1/access/avhrr/). The Argo data were obtained from the China Argo Real-Time Data Centre (CARDC, http://www.geodoi.ac.cn/WebEn/doi.aspx?Id=1823). These data were collected and made freely available by the International Argo Program and the national programs that contribute to it. (https://argo.ucsd.edu, https://www.ocean-ops.org). The Argo Program is part of the Global Ocean Observing System. CARS2009 Climatology data was provided by CSIRO Atlas of Regional Seas from www.cmar.csiro.au/cars. Global observation of nonlinear mesoscale eddies was provided by AVISO from https://www.aviso.altimetry.fr/en/data/products/value-added-products/global-mesoscale-eddy-trajectory-product/meta3-2-exp-nrt.html. CMEMS/DUACS surface geostrophic velocity data was obtained from https://data.marine.copernicus.eu/product/SEALEVEL_GLO_PHY_L4_MY_008_047/services. Ocean Surface Current Analysis Real-time (OSCAR) surface current data was available at http://apdrc.soest.hawaii.edu/dods/public_data/PODAAC/oscar_local/quarter_deg_v2.0_final. NCEP-DOE Reanalysis 2 daily air-sea heat flux data were obtained from https://psl.noaa.gov/data/gridded/data.ncep.reanalysis2.html. GLORYS12V1 mixed-layer depth data was provided by CMEMS from https://data.marine.copernicus.eu/product/GLOBAL_MULTIYEAR_PHY_001_030/description.

## Code availability

The code used to analyze the data and generate the results presented in this study can be obtained from https://github.com/scsio-ying/mhw^74^. The MatlabR2019a is used for plotting.
