# Supplementary material for: Vertical structures of marine heatwaves
Source: Nat Commun. 2023 Oct 14;14:6483. doi: 10.1038/s41467-023-42219-0 (PMC10576754; doi:10.1038/s41467-023-42219-0)
Supplement: Supplementary file 1 — Supplementary Information [file 41467_2023_42219_MOESM1_ESM.pdf]

Vertical structures of marine heatwaves

Supplementary Information

Ying Zhang[1], Yan Du[1,2]*, Ming Feng[3], Alistair J. Hobday[4]

[1]State Key Laboratory of Tropical Oceanography, South China Sea Institute of Oceanology, Chinese Academy of Sciences, Guangzhou, China.

[2]University of Chinese Academy of Sciences, Beijing, China.

[3]CSIRO Environment, Crawley, Western Australia, Australia

[4]CSIRO Environment, Hobart, Tasmania, Australia.

Corresponding author: Yan DU (duyan@scsio.ac.cn)

Supplementary Information

Supplementary Table 1

Supplementary Figures 1-7

Supplementary Table 1 | Recent prominent marine heatwaves (MHWs). The drivers and impacts of recent prominent MHWs are summarized from previous studies.

MHW-Location	MHW-Properties (Dur (days), Max (°C), Cum Intensity (°C))	MHW Drivers	MHW Impacts
Northeast Pacific (150°W-135°W, 40°N-50°N)	2013.11.17-2014.9.26 (314, 3.09, 633.50); 2015.1.3-2015.9.22 (263, 2.94, 474.84); 2019.5.22-2019.9.15(117, 2.89, 232.11); 2020.6.24-2020.9.29 (98, 2.77, 192.21)	Reduced loss of heat from the ocean to the atmosphere and weakened cold advection[1,2], associated with ocean-atmosphere interactions linked to tropical-extratropical teleconnections[3,4,5]	Massive mortality and reproductive failure of seabird (common murres[6]; Cassin's Auklets[7]); a decrease in net primary production and community production[8,9]; a coastwide bloom of toxigenic diatom Pseudo-nitzschia[10]; unprecedented and long-lasting decline of kelp forest[11,12]; shifts in pelagic micronekton and macrozooplankton community structure[13]; changes in biodiversity of pelagic forage species[14,15,16]; shifts in the horizontal and vertical distributions of marine species[15,17,18]; changes in biomass and distribution of fish stocks and potential fisheries catch (decrease in salmon and groundfish (cod, sablefish, and perch)[19]; humpback whales[15]); an unprecedented delay in the opening of the commercial Dungeness crab fishery in California[15]

East China Sea (118°E-130°E, 30°N-40°N)	2016.8.6-2016.8.26 (21, 2.74, 44.74); 2017.6.15-2017.6.26 (12, 1.22, 12.99); 2017.7.1-2017.8.12 (43, 2.55, 74.98); 2017.8.17-2017.8.29 (13, 1.75, 18.81); 2018.7.27-2018.8.16 (21, 2.30, 38.44)	Increased shortwave radiation related to a reduction of cloud cover, ocean advection carrying more warm water, weakened vertical mixing, and heat accumulated in an anticyclonic eddy[20]	Harmful algal blooms[21]; coral bleaching[22]; mass mortalities of farmed fish[23]
South China Sea (112°E-118°E, 8°N-22°N)	2020.5.30-2020.10.11 (135, 1.88, 176.83)	Weakened or even disappeared upwelling, northward shifted and weakened anticyclonic eddy and enhanced cyclonic eddy, weak eastward advective effect, and increased solar radiation and decreased latent heat release[24]	Coral bleaching[25,26]
Great Barrier Reef (143°E-156°E, 10°S-25°S)	2016.2.28-2016.4.16 (49, 1.47, 52.29); 2016.4.30-2016.8.12 (105, 1.61, 115.40)	Increased absorption of solar radiation due to reduced cloud cover, decreased turbulent heat release associated with reduced evaporative flux due to a moistened boundary layer[27,28]; shutdown of the North Queensland Coastal Current, and advection of warm water[28]; sub-thermocline upwelling variation[29]	Mass coral bleaching and mortalities[30,31,32]; declines in coral-feeding fishes; shifts in fish and invertebrate communities[33]
Tasman Sea (147°E-155°E, 37°S-45°S)	2015.9.9-2016.5.14 (249, 3.04, 451.95)	A strengthened southward extension of the East Australian	Oyster disease outbreaks; mollusks mortalities; cultured

		Current[34]	Atlantic salmon declines; range shift of fish species[34]; shift in abundance and composition of zooplankton community[35]
Peru (86°W-75°W, 0°-15°S)	2017.1.10-2017.4.1 (82, 2.70, 133.96)	Reduced latent heat release due to the wind relaxation, weakened wind-driven coastal upwelling, downwelling coastal waves induced-subsurface warming entraining into the mixed layer, and shoreward flow of warm surface waters[36]	Changes in rocky intertidal communities[37]; artisanal fisheries and scallop mariculture sectors were greatly affected[38]
West Tropical Indian Ocean (40°E-55°E, 5°S-5°N)	2015.8.1-2015.12.27 (149, 2.30, 246.54)	Downwelling waves induced-thermocline warming entraining into the mixed layer, horizontal advection of warm water [39,40]	Coral bleaching[41]; change in fish community structure[42]
Southeast Tropical Indian Ocean (100°E-120°E, 5°S-15°S)	2015.11.20-2016.12.1 (378, 1.83, 469.41)	Positive air-sea heat flux anomalies into the ocean[43], downwelling waves induced-subsurface warming entraining into the mixed layer[39,44]	Coral bleaching[45,46]; a decrease in small pelagic fish production[47]; Chl-a concentration and phytoplankton biomass increase slightly during atmospheric forcing-driven MHWs, while Chl-a concentration and phytoplankton biomass decrease dramatically during oceanic forcing-driven MHWs with a shift in phytoplankton community toward smaller-size species[44]
Southwest	2019.5.14-	Downwelling Rossby	No reported ecological

Tropical Indian Ocean (55°E-80°E, 8°S-18°S)	2020.2.15 (278, 1.64, 336.99); 2020.4.22-2020.9.21 (153, 1.54, 156.55)	waves induced-subsurface warming entraining into the mixed layer[39]	and socioeconomic impacts
Western Australia (110°E-116°E, 22°S-32°S)	2010.10.15-2011.4.9 (177, 4.19, 349.51)	A combination of a record strength Leeuwin Current and anomalously high air-sea heat flux into the ocean[48]	Loss of seagrass and kelp forests[49,50]; coral bleaching and mortality[51]; changes in biodiversity of temperate seaweeds, sessile invertebrates, and demersal fish[52]; mass mortality of Roei abalone, lobsters, fish, and major reductions in recruitment of scallops, king and tiger prawns, and blue swimmer crabs; and range shift of fish[53,54]
Mediterranean Sea (0-15°E, 36°N -42°N)	2003.5.2-2003.7.4 (33, 3.11, 76.35); 2003.7.8-2003.9.9 (64, 2.67, 128.57); 2014.9.8-2014.12.30 (114, 2.51, 177.55)	Anomalously clear skies and downward net radiative fluxes, strong surface stratification and low mixing[55,56,57]	Seagrass (Posidonia oceanica) shoot mortality[58]; mass mortality in rocky benthic communities[59,60]
Northwest Atlantic (76°W-68°W, 36°N-42°N)	2011.11.25-2012.6.4 (193, 4.11, 394.36)	Driven primarily by the anomalous air-sea heat flux, with a smaller contribution by the ocean advection[61,62]	Range shift of species, including commercially valuable fisheries species[63]
Western South Atlantic (55°W-35°W, 25°S-35°S)	2014.2.21-2014.5.19 (88, 1.43, 92.84); 2014.6.28-2014.8.23 (57, 1.43, 54.58); 2014.9.17-2015.1.30 (136,	Increased shortwave radiation due to reduced cloud cover and reduced ocean heat loss from weaker winds, associated with atmospheric blocking, and negligible	No reported ecological and socioeconomic impacts

	2.05, 170.64)	horizontal advection[64]	
Bengula (8°E-13°E, 12°S-20°S)	2001.4.14-2001.5.21 (38, 3.42, 104.86); 2010.12.14-2011.3.22 (99, 3.23, 237.95)	Oceanic downwelling induced- thermocline deepening led to an intensification of the Angola current and favored an intrusion of warm water[65,66]	No reported ecological and socioeconomic impacts

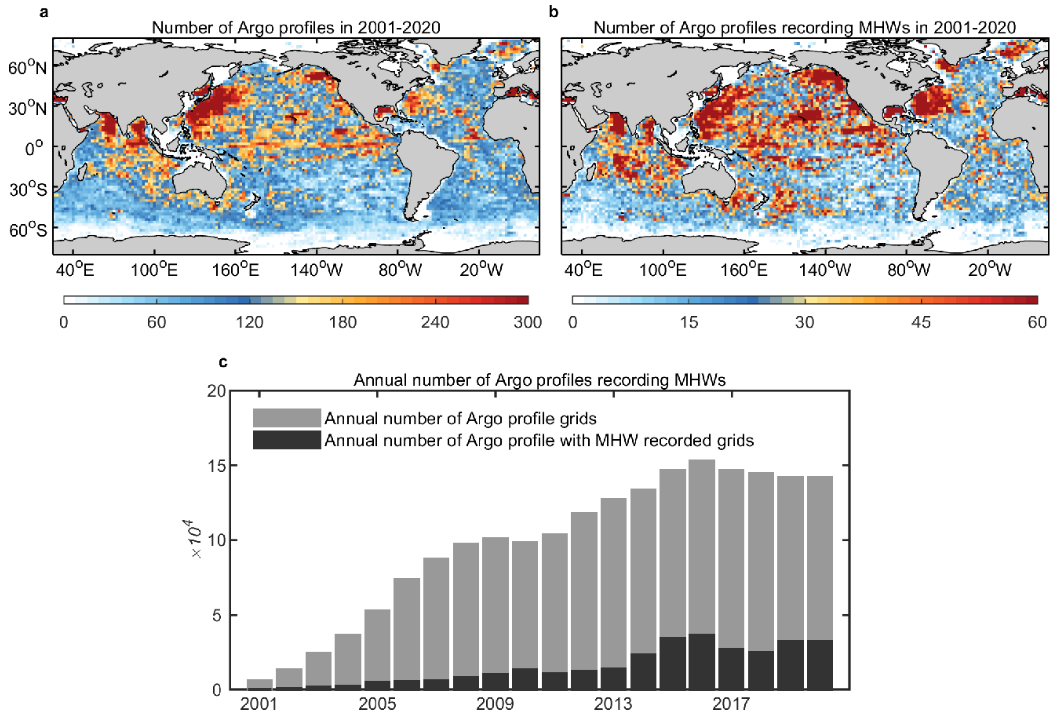

Supplementary Figure 1. Argo profiles and Argo profiles that recorded marine heatwaves (MHWs). a-b, Spatial distribution of the total number of Argo profiles and Argo profiles that recorded MHWs in 2001-2020, respectively. **c**, Globally integrated time series of the annual grid number of Argo profiles and Argo profiles that recorded MHWs. The bin size of grids is 2°×2°.

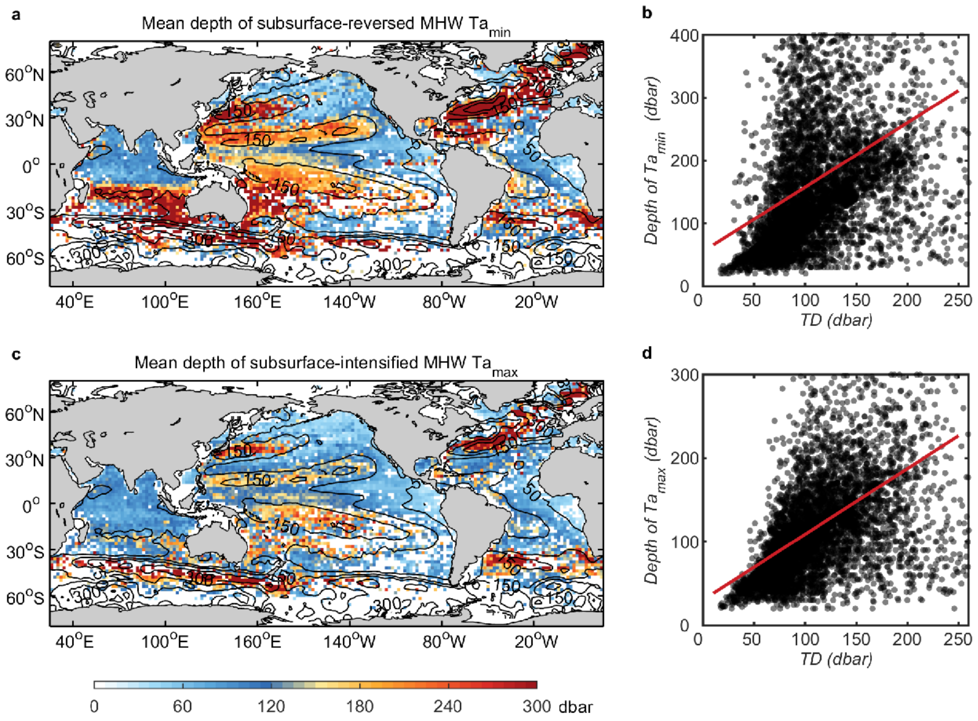

Supplementary Figure 2. Mean depth of the strongest cooling/warming signals (Ta_{min}/Ta_{max}) of subsurface-reversed/subsurface-intensified marine heatwaves (MHWs) and thermocline depth (TD). a, Mean depth of subsurface-reserved MHW Ta_{min} (shading, dbar) and TD (contours, dbar); **b**, Mean depth of subsurface-reserved MHW Ta_{min} vs. TD; **c**, Mean depth of subsurface-intensified MHW Ta_{max} (shading, dbar) and TD (contours, dbar); **d**, Mean depth of subsurface-intensified MHW Ta_{max} vs. TD.

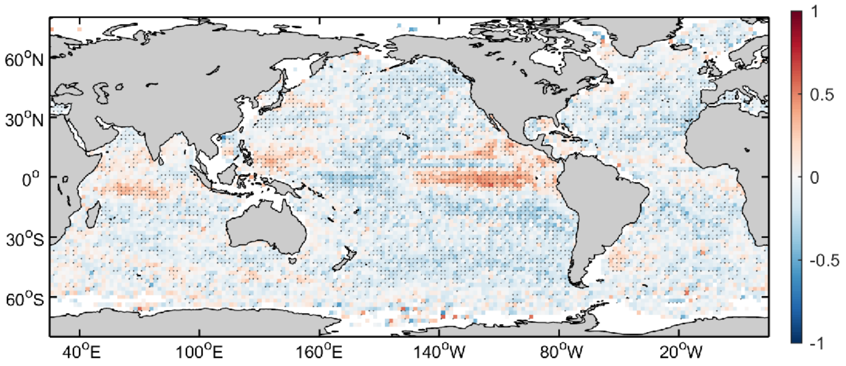

Supplementary Figure 3. Correlation between sea surface temperature anomalies and thermocline depth anomalies for the period 2001-2020. The dotted areas indicate the correlation coefficients are statistically significant at the 95% confidence levels based on a Student's *t*-test.

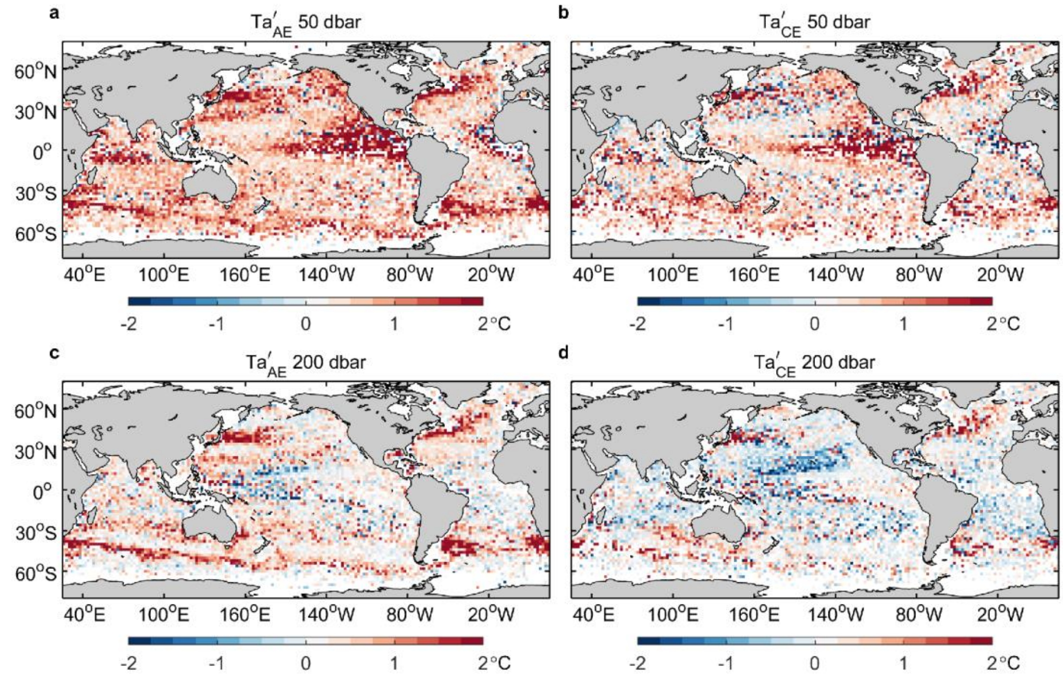

Supplementary Figure 4. Average of subsurface temperature anomalies induced by anticyclonic/cyclonic eddies (Ta'_{AE}/Ta'_{CE}) during marine heatwave events. a, temperature anomalies induced by anticyclonic eddies at 50 dbar. **b**, temperature anomalies induced by cyclonic eddies at 50 dbar. **e**, temperature anomalies induced by anticyclonic eddies at 200 dbar. **d**, temperature anomalies induced by cyclonic eddies at 200 dbar.

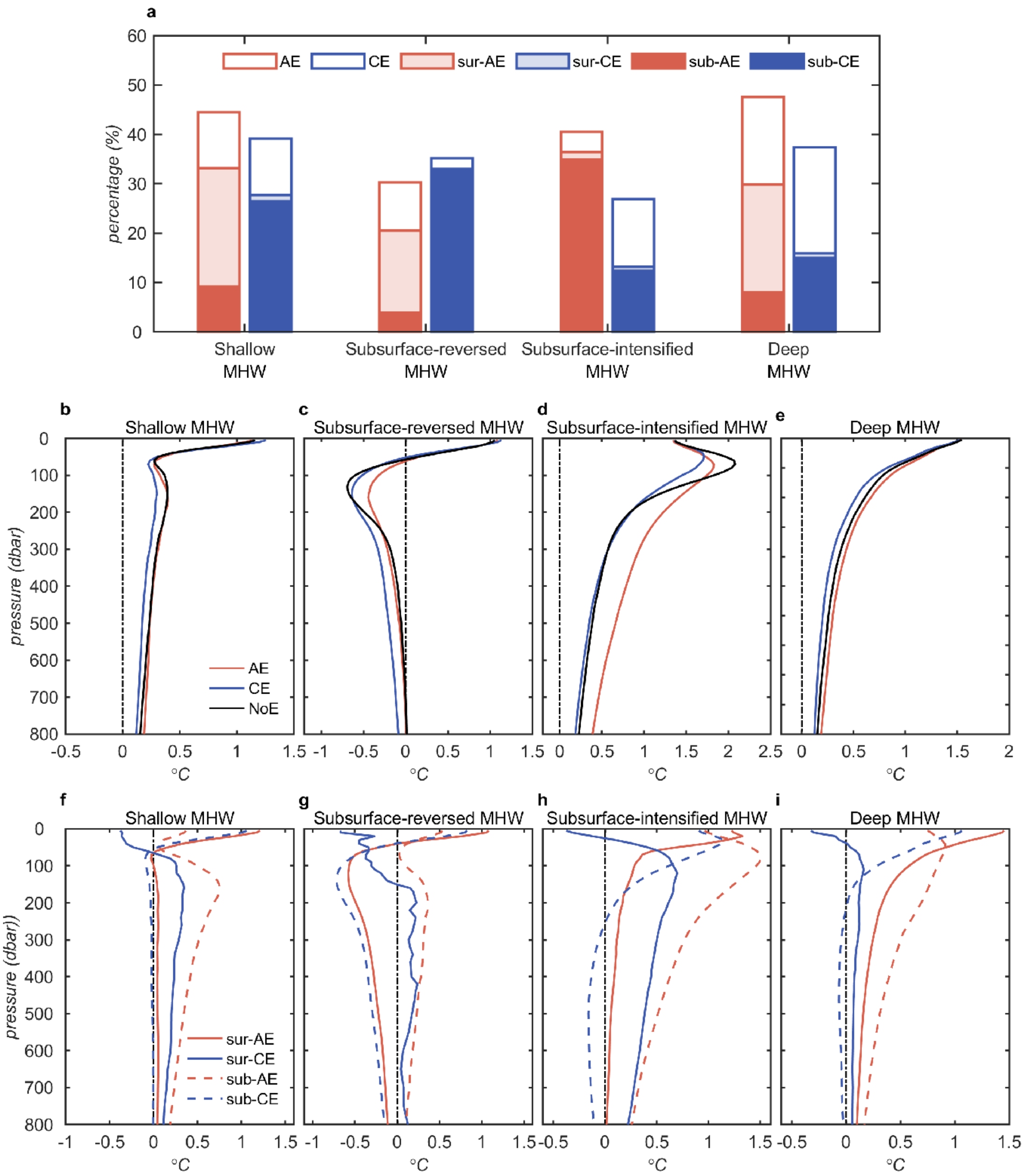

Supplementary Figure 5. Marine heatwaves (MHWs) occur with anticyclonic/cyclonic eddies. a, percentage of MHWs occurring with surface and subsurface anticyclonic/cyclonic eddies to the total MHWs of the global ocean. **b-e**, mean vertical temperature anomalies of global MHWs occurring with anticyclonic and cyclonic eddies (Ta_{AE} and Ta_{CE}), and without eddies (Ta). **f-i**, mean vertical temperature anomalies induced by surface/subsurface anticyclonic and cyclonic eddies (Ta'_{AE} and Ta'_{CE}) during MHW events. AE/CE denotes anticyclonic/cyclonic eddy, and NoE denotes no eddy. Sur-AE/CE denotes surface anticyclonic/cyclonic eddy, and sub-AE/CE denotes surface anticyclonic/cyclonic eddy.

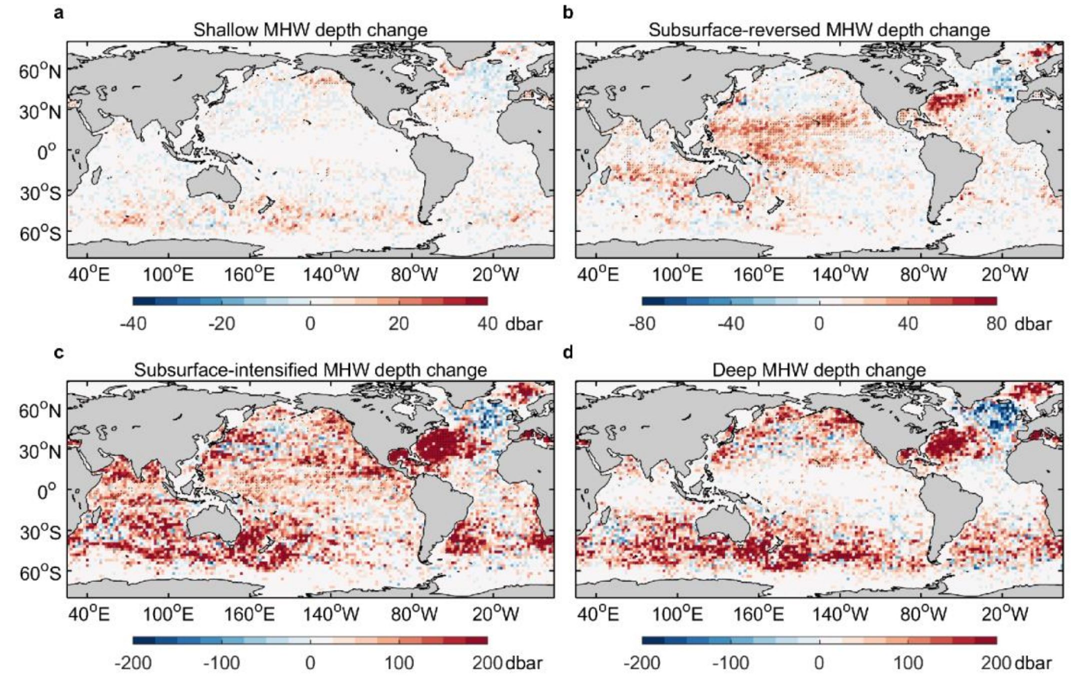

Supplementary Figure 6. Changes in impact depth of marine heatwaves (IDMHW) with different vertical structures between the periods 2001-2010 and 2011-2020 ($\overline{\mathbf{IDMHW}_{\mathbf{2011\text{-}2020}}} - \overline{\mathbf{IDMHW}_{\mathbf{2001\text{-}2010}}}$). **a**, shallow MHWs; **b**, subsurface-reserved MHWs; **c**, subsurface-intensified MHWs; **d**, deep MHWs. The dotted areas indicate the changes are statistically significant at the 95% confidence levels according to a Student's *t*-test.

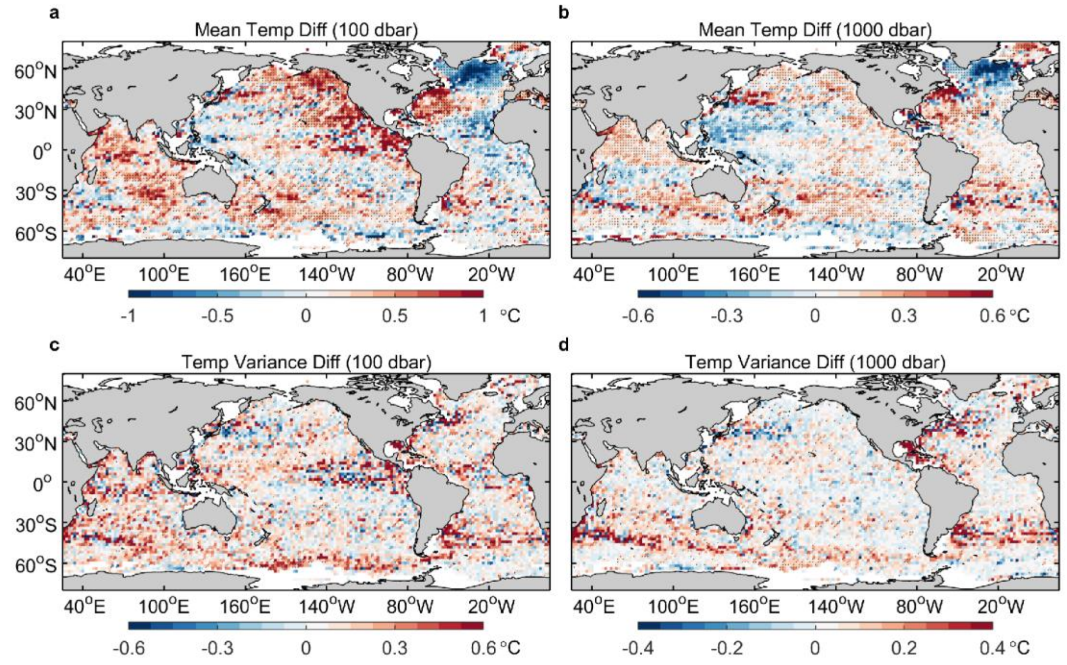

Supplementary Figure 7. Changes in Argo upper-ocean mean temperature and temperature variance between the periods 2001-2010 and 2011-2020 ($\overline{T_{2011\text{-}2020}} - \overline{T_{2001\text{-}2010}}$). **a**, mean temperature of the upper 100 dbar. **b**, mean temperature of the upper 1000 dbar. **c**, temperature variance of upper 100 dbar. **d**, temperature variance of upper 1000 dbar. The dotted areas indicate the changes are statistically significant at the 95% confidence levels according to a Student's *t*-test.

Supplementary References

1. Bond, N. A., Cronin, M. F., Freeland, H., & Mantua, N. Causes and impacts of the 2014 warm anomaly in the NE Pacific. *Geophys. Res. Lett.* **42,** 3414-3420 https://doi.org/10.1002/2015gl063306 (2015).
2. Gentemann, C. L., Fewings, M. R., & García-Reyes, M. Satellite sea surface temperatures along the West Coast of the United States during the 2014–2016 northeast Pacific marine heat wave. *Geophys. Res. Lett.* **44,** 312-319 https://doi.org/10.1002/2016GL071039 (2017).
3. Hartmann, D. L. Pacific sea surface temperature and the winter of 2014. *Geophys. Res. Lett.* **42,** 1894-1902 https://doi.org/10.1002/2015GL063083. (2015).
4. Di Lorenzo, E., & Mantua, N. Multi-year persistence of the 2014/15 North Pacific marine heatwave. *Nat. Clim. Chang.* **6,** 1042-1047 https://doi.org/10.1038/nclimate3082 (2016).
5. Hu, Z. Z., Kumar, A., Jha, B., Zhu, J., & Huang, B. Persistence and predictions of the remarkable warm anomaly in the northeastern Pacific Ocean during 2014–16. *J. Clim.* **30,** 689-702 https://doi.org/10.1175/JCLI-D-16-0348.1 (2017).
6. Piatt, J. F., et al. Extreme mortality and reproductive failure of common murres resulting from the northeast Pacific marine heatwave of 2014-2016. *PloS One* **15,** e0226087 https://doi.org/10.1371/journal.pone.0226087 (2020).
7. Jones, T., et al. Massive mortality of a planktivorous seabird in response to a marine heatwave. *Geophys. Res. Lett.* **45,** 3193-3202 https://doi.org/10.1002/2017GL076164 (2018).
8. Whitney, F. A. Anomalous winter winds decrease 2014 transition zone productivity in the NE Pacific. *Geophys. Res. Lett.* **42,** 428-431 https://doi.org/10.1002/2014GL062634 (2015).
9. Yang, B., Emerson, S. R., & Peña, M. A. The effect of the 2013–2016 high temperature anomaly in the subarctic Northeast Pacific (the "Blob") on net community production. *Biogeosciences* **15,** 6747-6759 https://doi.org/10.5194/bg-15-6747-2018 (2018).
10. McCabe, R. M., et al. An unprecedented coastwide toxic algal bloom linked to anomalous ocean conditions. *Geophys. Res. Lett.* **43,** 10-366 https://doi.org/10.1002/2016GL070023 (2016).
11. Arafeh-Dalmau, N., et al. Extreme marine heatwaves alter kelp forest community near its equatorward distribution limit. *Front. Mar. Sci.* **6,** 499 https://doi.org/10.3389/fmars.2019.00499 (2019).
12. Rogers-Bennett, L., & Catton, C. A. Marine heat wave and multiple stressors tip bull kelp forest to sea urchin barrens. *Sci. Rep.* **9,** 1-9 https://doi.org/10.1038/s41598-019-51114-y (2019).
13. Brodeur, R. D., Auth, T. D., & Phillips, A. J. Major shifts in pelagic micronekton and macrozooplankton community structure in an upwelling ecosystem related to an unprecedented marine heatwave. *Front. Mar. Sci.* **6,** 212 https://doi.org/10.3389/fmars.2019.00212 (2019).

14. Santora, J. A., et al. Impacts of ocean climate variability on biodiversity of pelagic forage species in an upwelling ecosystem. *Mar. Ecol. Prog. Ser.* **580,** 205-220 https://doi.org/10.3354/meps12278 (2017).
15. Santora, J. A., et al. Habitat compression and ecosystem shifts as potential links between marine heatwave and record whale entanglements. *Nat. Commun.* **11,** 1-12 https://doi.org/10.1038/s41467-019-14215-w (2020).
16. von Biela, V. R., et al. Extreme reduction in nutritional value of a key forage fish during the Pacific marine heatwave of 2014-2016. *Mar. Ecol. Prog. Ser.* **613,** 171-182 https://doi.org/10.3354/meps12891 (2019).
17. Li, L., et al. Subregional differences in groundfish distributional responses to anomalous ocean bottom temperatures in the northeast Pacific. *Global Change Biol.* **25,** 2560-2575 https://doi.org/10.1111/gcb.14676 (2019).
18. Cavole, L. M., et al. Biological impacts of the 2013–2015 warm-water anomaly in the Northeast Pacific: winners, losers, and the future. *Oceanography* **29,** 273-285 http://dx.doi.org/10.5670/oceanog.2016.32. (2016).
19. Cheung, W. W., & Frölicher, T. L. Marine heatwaves exacerbate climate change impacts for fisheries in the northeast Pacific. *Sci. Rep.* **10,** 1-10 https://doi.org/10.1038/s41598-020-63650-z (2020).
20. Gao, G., et al. Drivers of marine heatwaves in the East China Sea and the South Yellow Sea in three consecutive summers during 2016–2018. *J. Geophys. Res. Oceans* **125,** e2020JC016518 https://doi.org/10.1029/2020JC016518 (2020).
21. Lim, W., Go, W. J., Kim, K. Y., & Park, J. W. Variation in Harmful Algal Blooms in Korean coastal waters since 1970. *Journal of the Korean Society of Marine Environment & Safety*, **26,** 523-530 https://doi.org/10.7837/kosomes.2020.26.5.523 (2020).
22. Singh, T., Iijima, M., Yasumoto, K., & Sakai, K. Effects of moderate thermal anomalies on Acropora corals around Sesoko Island, Okinawa. *PloS One* **14,** e0210795 https://doi.org/10.1371/journal.pone.0210795 (2019).
23. Smith, K. E., et al. Socioeconomic impacts of marine heatwaves: Global issues and opportunities. *Science* **374,** eabj3593 https://doi.org/10.1126/science.abj3593 (2021).
24. Yao, Y., & Wang, C. Variations in summer marine heatwaves in the South China Sea. *J. Geophys. Res. Oceans* **126,** e2021JC017792 https://doi.org/10.1029/2021JC017792 (2021).
25. Feng, Y., et al. Marine heatwave events near Weizhou Island, Beibu Gulf in 2020 and their possible relations to coral bleaching. *Sci. Total Environ.* **823,** 153414 https://doi.org/10.1016/j.scitotenv.2022.153414 (2022).
26. Lyu, Y., Zhou, Z., Zhang, Y., Chen, Z., Deng, W., & Shi, R. The mass coral bleaching event of inshore corals form South China Sea witnessed in 2020: insight into the causes, process and consequence. *Coral Reefs* **41,** 1351-1364 https://doi.org/10.1007/s00338-022-02284-1 (2022).
27. Karnauskas, K. B. Physical diagnosis of the 2016 Great Barrier Reef bleaching event. *Geophys. Res. Lett.* **47,** e2019GL086177 https://doi.org/10.1029/2019GL086177 (2020).

28. Wolanski, E., Andutta, F., Deleersnijder, E., Li, Y., & Thomas, C. J. The Gulf of Carpentaria heated Torres Strait and the Northern Great Barrier Reef during the 2016 mass coral bleaching event. *Estuar. Coast. Shelf S.* **194,** 172-181 https://doi.org/10.1016/j.ecss.2017.06.018 (2017).
29. Frade, P. R., et al. Deep reefs of the Great Barrier Reef offer limited thermal refuge during mass coral bleaching. *Nat. Commun.* **9,** 3447 https://doi.org/10.1038/s41467-018-05741-0 (2018).
30. Hughes, T. P., et al. Global warming and recurrent mass bleaching of corals. *Nature* **543,** 373-377 https://doi.org/10.1038/nature21707 (2017).
31. Hughes, T. P., et al. Spatial and temporal patterns of mass bleaching of corals in the Anthropocene. *Science* **359,** 80-83 https://doi.org/10.1038/s41586-018-0041-2 (2018).
32. Eakin, C. M., Sweatman, H., & Brainard, R. E. The 2014–2017 global-scale coral bleaching event: insights and impacts. *Coral Reefs* **38,** 539-545 https://doi.org/10.1007/s00338-019-01844-2 (2019).
33. Stuart-Smith, R. D., Brown, C. J., Ceccarelli, D. M., & Edgar, G. J. Ecosystem restructuring along the Great Barrier Reef following mass coral bleaching. *Nature* **560,** 92-96 https://doi.org/10.1038/s41586-018-0359-9 (2018).
34. Oliver, E. C., et al. The unprecedented 2015/16 Tasman Sea marine heatwave. *Nat. Commun.* **8,** 1-12 http://dx.doi.org/10.1038/ncomms16101 (2017).
35. Evans, R., Lea, M. A., Hindell, M. A., & Swadling, K. M. Significant shifts in coastal zooplankton populations through the 2015/16 Tasman Sea marine heatwave. *Estuar. Coast. Shelf S.* **235,** 106538 https://doi.org/10.1016/j.ecss.2019.106538 (2020).
36. Echevin, V., et al. Forcings and evolution of the 2017 coastal El Niño off Northern Peru and Ecuador. *Front. Mar. Sci.* **5,** 367 https://doi.org/10.3389/fmars.2018.00367 (2018).
37. Valqui, J., et al. Changes in rocky intertidal communities after the 2015 and 2017 El Niño events along the Peruvian coast. *Estuar. Coast. Shelf S.* **250,** 107142 https://doi.org/10.1016/j.ecss.2020.107142 (2021).
38. Kluger, L. C., Kochalski, S., Aguirre-Velarde, A., Vivar, I., & Wolff, M. Coping with abrupt environmental change: The impact of the coastal El Niño 2017 on artisanal fisheries and mariculture in North Peru. *ICES J. Mar. Sci.* **76,** 1122-1130 https://doi.org/10.1093/icesjms/fsy171 (2019).
39. Zhang, Y., Du, Y., Feng, M., & Hu, S. Long-lasting marine heatwaves instigated by ocean planetary waves in the tropical Indian Ocean during 2015–2016 and 2019–2020. *Geophys. Res. Lett.* **48,** e2021GL095350. https://doi.org/10.1029/2021GL095350 (2021).
40. Qi, R., Zhang, Y., Du, Y., & Feng, M. Characteristics and drivers of marine heatwaves in the western equatorial Indian Ocean. *J. Geophys. Res. Oceans* **127,** e2022JC018732 https://doi.org/10.1029/2022JC018732 (2022).
41. Head, C. E., et al. Coral bleaching impacts from back-to-back 2015–2016 thermal anomalies in the remote central Indian Ocean. *Coral Reefs* **38,** 605-618 https://doi.org/10.1007/s00338-019-01821-9 (2019).

42. Samoilys, M. A., Halford, A., & Osuka, K. Disentangling drivers of the abundance of coral reef fishes in the Western Indian Ocean. *Ecol. Evol.* **9,** 4149-4167 https://doi.org/10.1002/ece3.5044 (2019).
43. Benthuysen, J. A., Oliver, E. C., Feng, M., & Marshall, A. G. Extreme marine warming across tropical Australia during austral summer 2015–2016. *J. Geophys. Res. Oceans* **123,** 1301-1326 https://doi.org/10.1002/2017JC013326 (2018).
44. Zhan, W., Zhang, Y., He, Q., & Zhan, H. Shifting responses of phytoplankton to atmospheric and oceanic forcing in a prolonged marine heatwave. *Limnol. Oceanogr.* **9999,** 1-4 https://doi.org/10.1002/lno.12388 (2023).
45. Babcock, R. C., et al. Severe continental-scale impacts of climate change are happening now: Extreme climate events impact marine habitat forming communities along 45% of Australia's coast. *Front. Mar. Sci.* **6,** 411 https://doi.org/10.3389/fmars.2019.00411 (2019).
46. Le Nohaïc, M., et al. Marine heatwave causes unprecedented regional mass bleaching of thermally resistant corals in northwestern Australia. *Sci. Rep.* **7,** 1-11 https://doi.org/10.1038/s41598-017-14794-y (2017).
47. Lumban-Gaol, J., et al. Impact of the strong downwelling (upwelling) on small pelagic fish production during the 2016 (2019) negative (positive) Indian Ocean Dipole events in the eastern Indian ocean off Java. *Climate* **9,** 29 https://doi.org/10.3390/cli9020029 (2021).
48. Pearce, A. F., & Feng, M. The rise and fall of the "marine heat wave" off Western Australia during the summer of 2010/2011. *J. Mar. Syst.* **112,** 139-156 https://doi.org/10.1016/j.jmarsys.2012.10.009 (2013).
49. Strydom, S., et al. Too hot to handle: Unprecedented seagrass death driven by marine heatwave in a World Heritage Area. *Global Change Biol.* **26,** 3525-3538 https://doi.org/10.1111/gcb.15065 (2020).
50. Wernberg, T., et al. Climate-driven regime shift of a temperate marine ecosystem. *Science* **353,** 169-172 https://doi.org/10.1126/science.aad8745 (2016).
51. Depczynski, M., et al. Bleaching, coral mortality and subsequent survivorship on a West Australian fringing reef. *Coral reefs* **32,** 233-238 https://doi.org/10.1007/s00338-012-0974-0 (2013).
52. Wernberg, T., et al. An extreme climatic event alters marine ecosystem structure in a global biodiversity hotspot. *Nat. Clim. Chang.* **3,** 78-82 https://doi.org/10.1038/NCLIMATE1627 (2013).
53. Pearce, A. F., et al. *The" marine heat wave" off Western Australia during the summer of 2010/11.* Fisheries Research Report No. 222 (p. 40). Department of Fisheries. (2011).
54. Caputi, N., et al. Management adaptation of invertebrate fisheries to an extreme marine heat wave event at a global warming hot spot. *Ecol. Evol.* **6,** 3583-3593 https://doi.org/10.1002/ece3.2137 (2016).
55. Black, E., Blackburn, M., Harrison, G., Hoskins, B., & Methven, J. Factors contributing to the summer 2003 European heatwave. *Weather* **59,** 217-223 https://doi.org/10.1256/wea.74.04 (2004).
56. Olita, A., et al. Effects of the 2003 European heatwave on the Central

Mediterranean Sea: surface fluxes and the dynamical response. *Ocean Sci.* **3,** 273-289 https://doi.org/10.5194/os-3-273-2007 (2007).

57. Darmaraki, S., Somot, S., Sevault, F., & Nabat, P. Past variability of Mediterranean Sea marine heatwaves. *Geophys. Res. Lett.* **46,** 9813-9823 https://doi.org/10.1029/2019GL082933 (2019).
58. Marbà, N., & Duarte, C. M. Mediterranean warming triggers seagrass (Posidonia oceanica) shoot mortality. *Global Change Biol.* **16,** 2366-2375 https://doi.org/10.1111/j.1365-2486.2009.02130.x (2010).
59. Garrabou, J., et al. Mass mortality in Northwestern Mediterranean rocky benthic communities: effects of the 2003 heat wave. *Global Change Biol.* **15**, 1090-1103 https://doi.org/10.1111/j.1365-2486.2008.01823.x (2009).
60. Garrabou, J., et al. Marine heatwaves drive recurrent mass mortalities in the Mediterranean Sea. *Global Change Biol.* **28,** 5708-5725 https://doi.org/10.1111/gcb.16301 (2022).
61. Chen, K., Gawarkiewicz, G. G., Lentz, S. J., & Bane, J. M. Diagnosing the warming of the Northeastern US Coastal Ocean in 2012: A linkage between the atmospheric jet stream variability and ocean response. *J. Geophys. Res. Oceans* **119,** 218-227 https://doi.org/10.1002/2013jc009393 (2014).
62. Chen, K., Gawarkiewicz, G., Kwon, Y. O., & Zhang, W. G. The role of atmospheric forcing versus ocean advection during the extreme warming of the Northeast US continental shelf in 2012. *J. Geophys. Res. Oceans* **120,** 4324-4339 https://doi.org/10.1002/2014jc010547 (2015).
63. Mills, K. E., et al. Fisheries management in a changing climate: lessons from the 2012 ocean heat wave in the Northwest Atlantic. *Oceanography* **26,** 191-195 http://dx.doi.org/10.5670/oceanog.2013.27. (2013).
64. Rodrigues, R. R., Taschetto, A. S., Sen Gupta, A., & Foltz, G. R. Common cause for severe droughts in South America and marine heatwaves in the South Atlantic. *Nat. Geosci.* **12,** 620-626 https://doi.org/10.1038/s41561-019-0393-8 (2019).
65. Rouault, M., Illig, S., Bartholomae, C., Reason, C. J. C., & Bentamy, A. Propagation and origin of warm anomalies in the Angola Benguela upwelling system in 2001. *J. Mar. Syst.* **68,** 473-488 https://doi.org/10.1016/j.jmarsys.2006.11.010 (2007).
66. Rouault, M., Illig, S., Lübbecke, J., & Koungue, R. A. I. Origin, development and demise of the 2010–2011 Benguela Niño. *J. Mar. Syst.* **188,** 39-48 https://doi.org/10.1016/j.jmarsys.2017.07.007 (2018).
